# Nitric Oxide Administration Using an Oxygen Hood: A Pilot Trial

**DOI:** 10.1371/journal.pone.0004312

**Published:** 2009-02-02

**Authors:** Namasivayam Ambalavanan, George T. El-Ferzli, Claire Roane, Robert Johnson, Waldemar A. Carlo

**Affiliations:** Department of Pediatrics, University of Alabama at Birmingham, Birmingham, Alabama, United States of America; Center for Research for Mothers & Children (CRMC), United States of America

## Abstract

**Background:**

We have shown earlier that inhaled nitric oxide (iNO) administered by oxygen hood reduces pulmonary hypertension in an animal model (J Perinatol 2002; 22:50-6). Our objective in this study was to determine feasibility of iNO by oxygen hood in neonates with elevated alveolar-arterial oxygen gradients (A-aDO_2_).

**Methods/Principal Findings:**

Masked randomized controlled pilot trial. Inclusion criteria were: gestation≥34 weeks, age<7 days, with post-ductal arterial line, and A-aDO_2_ 400–600. Infants were randomized to study gas (iNO 20 ppm or equivalent O_2_ flow) for 1 hr which was then weaned over the next 4 hours. Primary outcome was PaO_2_ one hour post-randomization. Four infants each were randomized to iNO or O_2_ (controls). Two of the four infants given iNO had an increase in PaO_2_ of >100 torr, while oxygenation was unchanged in the controls. Methemoglobinemia and other adverse effects were not noted in any infant. Environmental levels of NO and NO_2_ were minimal (<1 ppm) at >0.3 m from the hood.

**Conclusions:**

Administration of iNO by oxygen hood is feasible. Larger randomized controlled trials are required to measure the efficacy and determine an appropriate target population for this technique.

**Trial Registration:**

ClinicalTrials.gov NCT00041548

## Introduction

Severe neonatal pulmonary hypertension affects approximately 1 in 500 term neonates [1]. Large clinical trials have shown that inhaled nitric oxide (iNO) improves oxygenation in approximately 50% of infants who receive nitric oxide [2]. Analysis of results from three major trials [3]–[5] indicates that the use of extracorporeal membrane oxygenation (ECMO) decreased from 54% to 38% [6]. However, all controlled studies to date have studied nitric oxide administration to neonates who are already on mechanical ventilation. The use of iNO administered by oxygen hood has not been systematically evaluated.

Conventionally, neonates with hypoxemia and adequate respiratory effort are first adminstered oxygen by oxygen hood. If oxygenation is inadequate despite a high concentration of inspired oxygen (FiO_2_) or if respiratory acidosis occurs, the infant is placed on a mechanical ventilator. If oxygenation continues to be inadequate despite increased ventilator settings, rescue therapies such as iNO are attempted. However, mechanical ventilation by itself can lead to or potentiate lung injury and predispose to chronic lung disease, and high mean airway pressures may also decrease pulmonary blood flow and cardiac output. It is possible that earlier administration of iNO to neonates with abnormal gas exchange, rather than later as a rescue therapy, might accelerate the transition of the circulation from the fetal to neonatal physiology and improve oxygenation. This may in turn decrease the need for mechanical ventilation and its associated morbidity.

This study was designed as a pilot trial to evaluate the feasibility of NO administered by hood in neonates with elevated alveolar-arterial oxygen gradients (A-a DO_2_). Subsequent larger trials could determine if this method of NO administration can decrease the need for mechanical ventilation, ECMO, or other adverse outcomes.

## Methods

The protocol for this trial and supporting CONSORT checklist are available as supporting information; see Checklist S1 and Protocol S1.

Ethics statement: The study protocol was approved by the Institutional Review Board of the University of Alabama at Birmingham and parent(s) provided informed written consent before enrollment. Enrollment was done by the principal investigators (NA and WAC) or research coordinator (CR).

**Figure 1:**
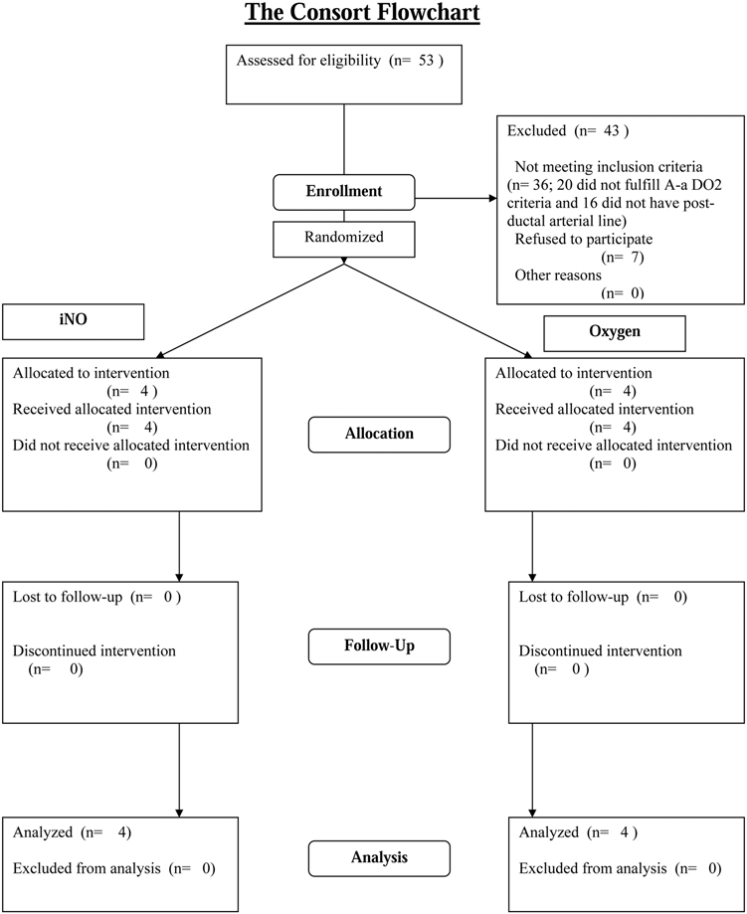
CONSORT flowchart

**Figure 2:**
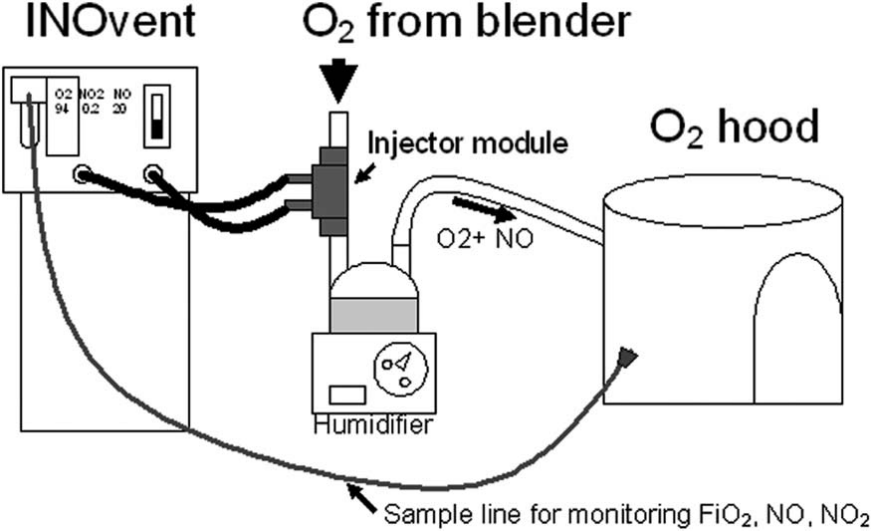
Delivery of inhaled nitric oxide into an oxygen hood using an INOvent®. The injector module from the INOvent® is inserted into the gas delivery circuit proximal to the humidifier (on the dry side). The sample line from the INOvent® measures the oxygen concentration, nitric oxide, and nitrogen dioxide in the oxygen hood.

Study design and protocol: This was a masked randomized controlled pilot trial on infants with elevated alveolar-arterial oxygen gradients (A-aDO_2_) admitted to the Regional Neonatal ICU of the University of Alabama at Birmingham, a regional perinatal center. The ClinicalTrials.gov Identifier for this study is NCT 00041548 (http://www.clinicaltrials.gov/ct2/show/NCT00041548). The CONSORT flowchart is shown as **Figure 1**. The inclusion criteria were: gestation ≥34 weeks at birth; age <7 days, post-ductal arterial line, and an A-aDO_2_ of 400 to 600 on two blood gases at least 30 minutes apart. After informed consent, infants were randomized using shuffled opaque envelopes to study gas (iNO at 20 ppm or equivalent flow of O_2_) for 1 hour. The gas was then weaned hourly over the next 4 hours (20 ppm→ 10 → 5 → 2.5 → 1 → off). It was planned that if a greater than 5% drop in oxygen saturation was observed during weaning, the study gas would be increased to the previous concentration and weaning would be done at 2 hourly intervals. No weaning of FiO_2_ was to be done while the study gas was being weaned. It was planned that if a greater than 5% drop in oxygen saturation or >5% methemoglobin was observed, the study gas would be rapidly weaned over 30 minutes and the infant would exit the study. The iNO was introduced into an oxygen hood (Oxydome ™ disposable hood; Maxtex® Inc.) using an INOvent (Datex-Ohmeda), using the arrangement as shown in **Figure 2**. The INOvent® was prepared by performing a low range (air) calibration and the standard pre-use procedures (leak test, system purge, and performance test). The INOvent® was connected to the oxyhood by placing the injector module inline on the dry side of the humidifier chamber. A minimum gas flow of 10 liters per minute of study gas was used to avoid accumulation of nitrogen dioxide (NO_2_) within the oxyhood. Monitoring of the three inspired gas values (O_2_, NO_2_, NO) was done by placing the end of the sample line inside the oxyhood. Due to mixing of exhaled gases inside the oxyhood, the “Set NO” was usually set 1–2 ppm higher to achieve the desired measured NO. If the baby was randomized to the control group, the INOmax® cylinder was opened and used only to pressurize the system, which prevented the “Low NO Pressure” alarm. A “Masking Shield” covered the Display/Control Panel and cylinder gauges, in order to maintain masking. Only the respiratory therapist and research coordinator was aware of the allocation assignment. Scavenging of the NO was not required as initial bench testing using 20 ppm of NO showed only trace amounts of NO (<1 ppm), comparable to routine use of iNO in mechanically ventilated neonates.

The primary outcome was the PaO_2_ one hour after the first hour of study gas. Secondary outcomes included: (1) A-a DO_2_ after one hour of exposure to treatment gas, (2) oxygen saturation by pulse oximetry (SpO_2_), (3) need for mechanical ventilation, (4) duration of oxygen therapy, (5) methemoglobin level in post-ductal arterial blood,* (6) Platelet count,* (7) Systemic blood pressure,* and (8) Environmental NO and NO_2_ exposure* (* Safety indicators).

The planned sample size for this study was 40, with 20 per group (nitric oxide or oxygen). The power of the study was 85% with n = 20 in each group, α = 0.05, assuming a difference in PaO_2_ of 25% with a standard deviation at 0.25 of mean. A non-parametric t-test was planned to evaluate the primary outcome of PaO_2_ after the first hour of study gas.

## Results

**Figure 3:**
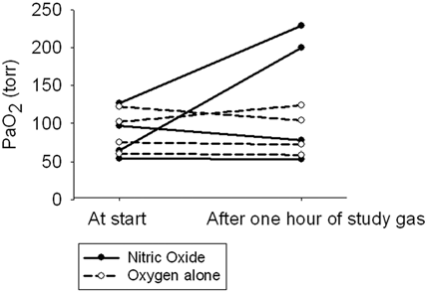
Change in PaO_2_ with study gas. Line graph showing change in PaO_2_ from before initiation of study gas (nitric oxide or oxygen) to one hour after study gas. Two of the four infants receiving nitric oxide (dark circles connected by solid lines) had an increase in PaO_2_ >100 torr, but none of the infants receiving O_2_ alone (open circles connected by dashed lines) demonstrated a significant change.

Eight term or near-term (36 or more completed weeks) appropriate for gestational age (2.5–4 kg birth weight) infants were enrolled over a three year period (May 2002–June 2005; **Figure 1**), and the study was terminated due to slow enrollment. Enrollment was slow as most infants transferred to our regional perinatal center for respiratory failure were already intubated either by the referring center or by our transport team. Four infants were white, five were female, and six were diagnosed with idiopathic persistent pulmonary hypertension of the newborn (PPHN), while one infant had early onset gram negative (E. coli) sepsis, and another had meconium aspiration syndrome (Table 1). All infants were receiving 90–95% O_2_ at enrollment. Four infants were randomized to iNO and four to O_2_ (controls). Two of the four infants given iNO had an increase in PaO_2_ of >100 torr, while oxygenation was essentially unchanged in the controls, although one patient had a small increase (+22 torr) (**Figure 3**, Table 1). The median PaO_2_ after the first hour of study gas in the infants receiving iNO was 139 torr (25^th^–75^th^ centile: 66–215 torr) versus 88 torr (66–114) in the infants receiving O_2_ (controls) (p NS). However, the small sample size indicates that statistical analysis would not be useful in this pilot study due to lack of statistical power. No methemoglobinemia (all levels <2% in both groups), hypotension (drop in systemic blood pressure >10%), thrombocytopenia/excessive bleeding or other adverse effects were noted in any of the infants. No “rebound phenomenon” was noted on weaning of the study gas. NO_2_ within the hood was less than 1 ppm in all infants over the duration of the study. Environmental levels of NO and NO_2_ were minimal (<1 ppm) to undetectable at >0.3 m from the hood at the bedside.

**Table 1:** Characteristics of neonates enrolled in the trial, and their response to the study gas.

Group	Diagnosis	Initial A-aDO2	Final A-aDO2	Initial PaO2 (torr)	Final PaO2 (torr)	Change in PaO2	Pre-trial O2	Post-trial O2	Need for IMV	Length of Stay (days)
O_2_-1	Sepsis	492	479	102	124	+22	2 days	4 days	No	14
O_2_-2	PPHN	586	587	75	72	−3	2 days	4 days	No	7
O_2_-3	PPHN	551	568	122	104	−18	2 days	11 days	Yes	26
O_2_-4	PPHN	580	581	61	59	−2	20 h	4 days	Yes	10
NO-1	Mec Asp	564	428	64	200	+136	5 h	24 h	No	5
NO-2	PPHN	561	575	97	78	−19	24 h	9 days	Yes	12
NO-3	PPHN	554	585	54	53	−1	4 h	6 days	No	14
NO-4	PPHN	523	422	127	229	+102	33 h	6 days	Yes	14

## Discussion

This pilot trial demonstrates the technique and feasibility of iNO administration using an oxygen hood and a commercial iNO delivery device. This trial had an inadequate sample size to demonstrate efficacy or safety. However, the efficacy and safety of iNO in intubated infants is already known [2]–[6], and the purpose of this pilot trial was to show if iNO could be administered into an oxygen hood in the clinical setting. Two of the four infants who received iNO had an increase in PaO_2_ >100 torr, while none of the infants in the oxygen control groups had a similar increase. Neonates with hypoxemia (PaO_2_ <50 torr) or respiratory acidosis while on 100% oxygen at the onset are usually intubated and mechanically ventilated. The A-a DO_2_ usually is >600 torr in this situation, and often more than 630 torr, depending on the PaCO_2_. However, neonates who present with markedly elevated A-a DO_2_ (>400 torr) but with adequate oxygenation (PaO_2_ 50–150 torr on FiO_2_ ≥0.95), respiratory effort, and acid-base status often present a dilemma – to intubate and mechanically ventilate or not? These neonates with either a severe V/Q mismatch (intra-pulmonary shunt) or right to left shunts (extra-pulmonary shunt) (frequently both mechanisms may be operative to a variable degree) are often maintained on high oxygen concentrations (FiO_2_ >0.7) delivered by oxygen hood, in an attempt to maintain adequate oxygenation and possibly accelerate the spontaneous resolution of elevated pulmonary pressures and minimize the risk of hypoxic pulmonary vasoconstriction or V/Q mismatch without resorting to mechanical ventilation. While some of these neonates improve spontaneously, others persist with high A-a DO_2_ and ultimately require mechanical ventilation and occasionally ECMO. It is possible that these infants may benefit from iNO by oxygen hood.

Since the observation that iNO can cause selective pulmonary vasodilation in neonates [7], [8], multiple randomized controlled trials [3]–[6] have been performed and a systematic review indicates that iNO may benefit neonates with hypoxemic respiratory failure [2]. The majority of the available literature as briefly described above deals with neonates on mechanical ventilation, since NO has been used so far as a “rescue” therapy for infants not responding to conventional, and in some cases, high frequency ventilation. The current practice of intubation and mechanical ventilation of the majority of infants with hypoxemic respiratory failure makes it difficult to evaluate non-invasive methods of iNO delivery such as by oxygen hood. iNO has also been delivered by nasopharyngeal tube [9], pulsed nasal cannula, and face mask [10]. However, due to entrainment of room air and varying contributions from mouth breathing, the actual delivered NO level by nasal or nasopharyngeal cannulae cannot be ascertained with accuracy. Especially during weaning, it is important that the iNO be reduced gradually and carefully controlled in order to avoid rebound pulmonary hypertension. The delivered oxygen and iNO concentrations may possibly be regulated better with the oxygen hood than with the nasal cannulae. The other problem with nasal cannulae is that the presence of a nasal prong(s) will partially occlude the nostrils and increase airway resistance significantly, further compromising respiratory effort and oxygenation in a neonate with abnormal gas exchange.

A major proportion of morbidity and mortality in term neonates is due to hypoxemic respiratory failure. Fortunately, the incidence of hypoxic respiratory failure in newborn infants has decreased in recent years, but this has led to poor accrual for clinical trials on PPHN [11]. A limitation of our pilot trial in a regional perinatal center is that many potential subjects were transported already intubated. Randomized multi-center clinical trials involving large delivery centers are required to determine if non-invasive administration of iNO into an oxygen hood decreases the need for mechanical ventilation and subsequent need for ECMO in this population, and if this improved short-term outcome results in a lower mortality, earlier discharge and both direct and indirect cost savings.

## Supporting Information

Checklist S1 CONSORT Checklist
(0.06 MB DOC)

Protocol S1 Trial protocol
(0.11 MB DOC)
