# Supplementary material for: Nitric Oxide Administration Using an Oxygen Hood: A Pilot Trial
Source: PLoS One. 2009 Feb 2;4(2):e4312. doi: 10.1371/journal.pone.0004312 (PMC2629563; doi:10.1371/journal.pone.0004312)
Supplement: Protocol S1 — Trial protocol (0.11 MB DOC) [file pone.0004312.s002.doc]

# **SUMMARY**

Inhaled nitric oxide in neonates with elevated A-a DO_2_ gradients not requiring mechanical ventilation

**Objective:** This is a pilot study to evaluate whether administration of nitric oxide gas by oxygen hood at 20 ppm significantly increases PaO_2_, as compared to placebo gas (oxygen), within one hour of initiation, and with no significant adverse effects.

**Study design:** Single center, masked, placebo-controlled randomized crossover trial

**Sample Size:** Total 40 (two groups of 20 each)

**Eligibility criteria:**

*Inclusion criteria:*

1. Gestational Age >34 weeks

2. Age < 48 hrs

3. A-a DO_2_ >500, <600 on two arterial blood gases one hour apart, while on 100% O_2_ by oxygen hood

4. Arterial access (post-ductal)

5. Admitted to UAB Regional NICU (Inborn or Outborn)

*Exclusion criteria:*

1. Cardiac disease other than PDA or PFO
2. Rapid deterioration requiring mechanical ventilation before entry into the study
3. Major malformations
4. Major neurologic or metabolic disorder or other illness leading to hypoventilation and hypercarbia

**Study intervention:** After obtaining informed consent, neonates will be randomized to either placebo (oxygen) or NO 20 ppm. The treatment gas will be administered in the inner hood of a double hood system. FiO_2_ and NO/NO_2_ concentrations will be monitored continuously. Scavenging will be done by a suctioning apparatus in the outer hood. Respiratory therapists will adjust flow and blending to administer 20 ppm of NO or an equivalent flow of O_2_. Physicians and nurses caring for the patient and analyzing results will be blinded to patient assignment. After completion of one hour on the study, the gas will be weaned over the next four hours, then discontinued. If at the end of the study period the infant still meets entry criteria, he/she will be crossed over into the other treatment group. The attending physician can initiate mechanical ventilation at any point during this study, based on clinical or other grounds.

**Study measurements:** Arterial blood gases (ABGs; post-ductal) will be obtained just before initiation (0 hrs) and every hour for the next six hours. ***The PaO_2_ after exposure to one hour of the treatment gas will be the primary outcome measure***. Continuous pulse oximetry will also be performed. Assessment will be made for any “rebound phenomenon” in oxygenation and any adverse effects, including methemoglobinemia, bleeding tendencies, and thrombocytopenia (platelet count before and after the study).

**Sample size estimate:** Repeated measures ANOVA or unpaired t-test will be used if data are normally distributed, otherwise non-parametric tests will be used. The power of the study is 85% with n=20 in each group, α=0.05, assuming we need to detect a difference in PaO_2_ of 25% with a standard deviation at 0.25 of mean. Small numbers are required since this is a pilot study to demonstrate physiologic efficacy and safety, and is not a study to demonstrate effect on clinical outcomes.

**Inhaled nitric oxide in neonates with elevated A-a DO2 gradients not requiring mechanical ventilation**

## Namasivayam Ambalavanan MD

Elaine St. John MD
Waldemar Carlo MD

University of Alabama at Birmingham

## Contents

1. Introduction and hypotheses
   1. Primary outcome measure
   2. Secondary outcome measures
   3. Hypotheses
2. Background and significance
   1. Elevated A-a DO_2_ gradients in neonates
   2. Use of nitric oxide in neonates
   3. Clinical significance of proposed protocol
3. Experimental design and methods
   1. Design
   2. Entry and exclusion criteria
   3. Randomization procedure
   4. Apparatus, protocols, and monitoring
   5. General care
   6. Co-interventions and contamination
   7. Study exit criteria
   8. Outcomes and definitions
   9. Statistical issues
4. Ethical considerations
5. Feasibility
   1. Available population / estimated recruitment time
   2. Potential pitfalls of study design
6. References
7. Appendices

### A: Consent form

B: Data collection form

1. **Introduction and hypotheses**

Severe neonatal pulmonary hypertension continues to affect approximately one in 1000 term neonates, with a mortality rate of >50% in the absence of extracorporeal membrane oxygenation (ECMO), and 10-20% even with the availability of ECMO. Morbidity consisting of severe handicap, intracranial hemorrhage, or deafness, continues to occur in more than 20% in survivors. The most common cause is a maladaptation of the neonatal pulmonary circulation to the extra-uterine environment, which may be either idiopathic or induced by sepsis, meconium aspiration, or asphyxia. Nitric oxide (NO), a vasodilator, can be administered by inhalation to produce effects largely limited to the pulmonary circulation. NO inhalation has undergone multiple phase I and II trials, as well many large multicenter clinical trials in neonates with encouraging results. Analysis of results from three major trials (1-3) indicate that the use of ECMO decreased from 54% to 38%, with no prolongation of total ventilator treatment, hospital stay, or incidence of chronic lung disease (4). However, all controlled studies to date have studied nitric oxide administration to neonates who are already on mechanical ventilation.

Conventionally, neonates with hypoxemia and adequate respiratory effort are first adminstered oxygen by oxygen hood. If oxygenation is inadequate despite a high concentration of inspired oxygen (FiO_2_) or if respiratory acidosis occurs, the infant is placed on a mechanical ventilator. If oxygenation continues to be inadequate despite increasing ventilator settings, rescue therapies such as inhaled nitric oxide or high frequency ventilation are attempted. Lack of response to these therapies usually results in initiation of ECMO. One of the problems with this plan of management is that mechanical ventilation by itself can lead to or potentiate lung injury and predispose to chronic lung disease. The high mean airway pressures that are often required may also decrease pulmonary blood flow and cardiac output. It is possible that administration of inhaled NO to neonates with abnormal gas exchange earlier, rather than later as a rescue therapy in a moribund state, might accelerate the transition of the circulation from the fetal to neonatal physiology and improve oxygenation. This may in turn decrease the need for mechanical ventilation, its associated morbidity, and perhaps even ECMO.

This study is designed as a pilot study to evaluate the physiologic efficacy (rather than effect on clinical outcomes) of NO administered by hood in improving oxygenation of neonates with elevated alveolar - arterial oxygen gradients (A-a DO_2_). If this is shown to be achievable, subsequent larger trials can determine if this method of NO administration can decrease the need for mechanical ventilation, ECMO, or other major outcomes.

1.1 Primary outcome measure:

Partial pressure of oxygen in post-ductal arterial blood (PaO_2_)

1.2 Secondary outcome measures:

1. Alveolar-arterial oxygen gradient (A-a DO_2_) after one hour of exposure to treatment gas
2. Alveolar-arterial oxygen ratio (A/a O_2_)
3. Oxygen saturation by pulse oximetry (SpO_2_)
4. Need for mechanical ventilation
5. Duration of oxygen therapy
6. Methemoglobin level in post-ductal arterial blood (MetHb)*
7. Platelet count*
8. Systemic blood pressure*
9. Environmental NO and NO_2_ exposure*

(* Safety indicators)

1.3 Hypotheses

The primary hypothesis is that administration of nitric oxide gas by oxygen hood at 20 ppm to neonates with elevated A-a DO_2_ (500-600 mmHg) significantly increases PaO_2_, as compared to placebo gas (oxygen), within one hour of initiation, and with no significant adverse effects.

Secondary hypotheses

1. Administration of nitric oxide decreases A-a DO_2_as compared to placebo gas (oxygen)
2. Administration of nitric oxide decreases A/a O_2_ as compared to placebo gas (oxygen)
3. Administration of nitric oxide increases SpO_2_ as compared to placebo gas (oxygen)
4. Administration of nitric oxide decreases the need for mechanical ventilation as compared to placebo gas (oxygen)
5. Administration of nitric oxide at 20 ppm by oxygen hood does not increase duration of oxygen therapy significantly as compared to placebo gas (oxygen)
6. Administration of nitric oxide at 20 ppm by oxygen hood does not increase MetHb significantly as compared to placebo gas (oxygen)
7. Administration of nitric oxide at 20 ppm by oxygen hood does not decrease platelet counts significantly as compared to placebo gas (oxygen)
8. Administration of nitric oxide at 20 ppm by oxygen hood does not decrease mean systemic blood pressure significantly as compared to placebo gas (oxygen)
9. Administration of nitric oxide at 20 ppm by oxygen hood does not increase environmental NO and NO_2_ significantly as compared to placebo gas (oxygen)

# **2. Background and significance**

2.1 Elevated A-a DO_2_ gradients and impaired oxygenation in neonates

The A-a DO_2_ gradient indicates the difference between the partial pressure of oxygen in the alveoli and that in the arterial blood. This is normally about 10 mmHg in adults and 25 mmHg in neonates after the first two hours of life. At birth there is a transition from the fetal circulation to the neonatal circulation, characterized by a rapid drop in pulmonary vascular resistance with the onset of ventilation, accompanied by improvement in oxygenation. The efficiency of gas exchange in the lungs can be indicated by the ratio of ventilation to perfusion (V/Q). Even in the normal newborn, the distribution of ventilation and perfusion is not as optimal as in older infants or children. A large proportion of the pulmonary blood flow is directed to atelectatic or poorly ventilated alveoli or is shunted right to left through the ductus arteriosus (normally open in the first few hours of life) (5). This results in an elevated A-a DO_2_ and decreased PaO_2_ for the neonate as compared to the adult. The A-a DO_2_ may be as high as 35 mm Hg in the first two hours of life, decreasing to 25 mmHg as lung volume and compliance improve (of the A-a DO_2_ of 25 mm Hg in the neonate, 14 mm Hg are the normal shunt component and 10 mmHg are the distribution component, with a dead space component of 1 mm Hg and a diffusion component of <1 mm Hg). The normal PaO_2_ in room air is hence > 60 mm Hg by 2 hours of age and > 70 mm Hg by 12-24 hours of age.

#### A high A-a DO_2_ hence results from V/Q mismatch or from right to left shunting, either intra-pulmonary or intra-cardiac (shunts are extreme cases of V/Q mismatch where V/Q = 0). This can result from either pulmonary disease, such as with meconium aspiration syndrome, or with elevation of pulmonary pressures to systemic or supra-systemic levels, as with sepsis or idiopathic pulmonary hypertension. The hyperoxia test is often used in term and near-term neonates to differentiate between the right to left shunt as in heart disease or PPHN and a ventilation-perfusion mismatch. A neonate with a mild or moderate V/Q mismatch would usually have a PaO_2_ > 100 mm Hg after exposure for 5 to 10 minutes to 100% oxygen supplied by a hood, due to diffusion of oxygen to poorly ventilated alveoli. Hyperventilation in combination with hyperoxia may help to further differentiate the fixed right to left shunt of heart disease from labile PPHN.

#### Neonates with hypoxemia (PaO_2_ < 50 mm Hg) or respiratory acidosis while on 100% oxygen at the onset are usually intubated and mechanically ventilated. The A-a DO_2_ usually is > 600 mm Hg in this situation, and often more than 630 mm Hg, depending on the PaCO_2_. However, neonates who present with markedly elevated A-a DO_2_ (> 500 mm Hg) but with adequate oxygenation (PaO_2_ 50-150 mm Hg on FiO_2_ > 0.95), respiratory effort, and acid-base status often present a dilemma – to intubate and mechanically ventilate or not? These neonates with either a severe V/Q mismatch (intra-pulmonary shunt) or right to left shunts (extra-pulmonary shunt) (frequently both factors are operative to a variable degree) are often maintained on high oxygen concentrations (FiO_2_ > 0.7) delivered by oxygen hood, in an attempt to maintain adequate oxygenation and possibly accelerate the spontaneous resolution of elevated pulmonary pressures and minimize the risk of hypoxic pulmonary vasoconstriction or V/Q mismatch without resorting to mechanical ventilation. While some of these neonates improve spontaneously, others persist with high A-a DO_2_ and ultimately require mechanical ventilation and occasionally ECMO. The neonates who deteriorate are often those with worse oxygenation and higher A-a DO_2_.

2.2 Inhaled nitric oxide in neonates

Since the discovery that NO is the endothelium derived relaxing factor (6), and the observation that inhaled NO can cause selective pulmonary vasodilation in neonates (7,8), it has been hoped that inhaled NO would be the “magic bullet” that would help manage PPHN. The early case series of Roberts et al. (7) and Kinsella et al. (8) in 1992 showed promising effects of inhaled NO in improving oxygenation without causing systemic hypotension or causing methemoglobinemia. Day et al. (9) randomized neonates with oxygenation indices (OI) of 25 to 40 to receive conventional therapy with or without 20 ppm NO, while neonates with OI >40 all received NO. Systemic hemodynamics did not change while blood gases and ductal shunting acutely improved only in those patients treated with NO. Finer et al. (10) evaluated the dose response to inhaled NO in 23 infants. Eleven of 13 infants with documented evidence of PPHN responded with an increase of PaO_2_ >10 mm Hg or SpO2>10%. No significant difference was noted in the PaO_2_ or A-a DO_2_ response to inhaled NO between 5 and 80 ppm. While these initial studies focused on physiologic outcomes, many subsequent single-center and multi-center trials examined the effect of NO on clinical outcomes. Barefield et al. (11) also showed that inhaled NO led to transient improvements in oxygenation. However, in this study, inhaled NO did not reduce the incidence of meeting ECMO criteria. In a larger trial, Wessel et al. (1) randomized 49 mechanically ventilated neonates with proven PPHN to treatment with or without NO. Sustained improvement in oxygenation was seen in the NO group (the median percentage change for PaO_2_ was +43% in the NO group and –2% in the control group), although this did not result in decreased mortality or use of ECMO. Only one patient with alveolar capillary dysplasia developed methemoglobinemia after 25 hours of treatment with 80 ppm of NO. None of the patients with favorable transient responses later deteriorated to require ECMO support. Roberts et al. (2) in a multicenter study (the Inhaled Nitric Oxide study group) randomized 58 term neonates with severe hypoxemia and PPHN to either a mixture of oxygen with either a control gas (nitrogen) or NO (80 ppm). If a response to NO was seen within 20 minutes, the treatment was considered successful and was continued at lower concentrations. NO doubled PaO_2_ in 53%, while only 7% of controls improved. ECMO was required in 71% of the control group and only 40% of the NO group (p=0.02). NO did not cause systemic hypotension, methemoglobinemia, or other side effects. The NINOS study group (3) also conducted a larger (n=235) multicenter study of inhaled NO in neonates >34 weeks gestation with an OI>25. In contrast to the previous study, oxygen was used as the control gas rather than nitrogen. The combined outcome of mortality or need for ECMO was lower in the NO group (46% in NO group vs. 64% in control group, p<0.006). The need for ECMO was also lower (NO group 39% vs. control group 54%, p=0.01), with the NO group showing significant improvement in oxygenation without evidence of toxicity. In a recent commentary, Truog (4) analyzed the cumulative total of the neonates in the previous three studies (1-3). While noting that NO decreased the use of ECMO from 54 to 38%, without increasing the length of stay or prolongation of mechanical ventilation, he also noted that 13% of the enrolled infants died despite all available therapy and that 40% of the neonates did not respond to even high doses of 40-80 ppm of NO (4).

The majority of the available literature as briefly described above deals with neonates on mechanical ventilation, since NO has been used so far as a “rescue” therapy for infants not responding to conventional, and in some cases, high frequency ventilation. Kakuya et al. (12) reported on the use of inhaled NO via a nasopharyngeal tube in an infant with a severely hypoplastic lung and end-stage pulmonary hypertension. Clinical improvement was maintained for seven days with 18-22 ppm NO inhalation. Environmental NO levels were less than 0.06 ppm and NO_2_ < 0.3 ppm throughout the treatment, well within safety margins. The use of a nasopharyngeal NO delivery system without sedation, as an alternative to endotracheal intubation and sedation, was thought to be advantageous and a practical method in this situation for maintaining a certain quality of life for the patient and family. However, due to entrainment of room air with varying patterns of respiration and varying contributions from mouth breathing, the actual delivered NO level by this method cannot be ascertained with accuracy. The other problem with this method is that the presence of a nasal catheter will partially or even completely occlude one nostril and increase airway resistance significantly (possibly by up to 50%) (13,14), further compromising respiratory effort and oxygenation in a neonate with abnormal gas exchange. NO has also been delivered by a pulsed nasal cannula in older infants and children and shown to decrease pulmonary arterial pressures and resistance as effectively as NO delivered by face mask (15). However, this method has the same flaws as delivery with a nasopharyngeal cannula (12).

2.3 Clinical significance of proposed protocol

A major proportion of morbidity and mortality in term neonates is due to respiratory disorders characterized by abnormal gas exchange and elevated A-a DO_2_. Simple non-invasive techniques of NO administration may decrease the need for mechanical ventilation and subsequent need for ECMO in this population. This improved short-term outcome may result in a lower mortality, earlier discharge and both direct and indirect cost savings. None of the studies to date have so far evaluated NO administration by oxygen hood in the neonatal population, though NO administration during mechanical ventilation has been shown to be effective and preliminary data on alternative methods of administration such as by nasal and nasopharyngeal cannulae have been shown to be feasible. Therefore, this protocol has been designed as a pilot trial with sufficient power to evaluate the physiologic efficacy of inhaled NO in improving oxygenation in neonates with markedly elevated A-a DO_2_ gradients.

**3. Experimental design and methods**

3.1 Design

This clinical trial will be a randomized and placebo-controlled crossover single center study.

3.2 Entry and exclusion criteria

*Entry criteria:*

1. Gestational Age >34 completed weeks (>35)

2. Age < 48 hrs

3. A-a DO_2_ >500, <600, on two post-ductal arterial blood gases one hour apart, while on 100% O_2_ by oxygen hood

1. Post-ductal arterial access (UAC or posterior tibial or dorsalis pedis arterial line)
2. Admitted to UAB Regional NICU (Inborn or Outborn)

*Exclusion criteria:*

### 1. Cardiac disease (structural disease with right to left or mixing lesions), not including patent ductus arteriosus (PDA) or patent foramen ovale (PFO)

2. Rapid deterioration requiring mechanical ventilation before entry into the study

3. Major malformations

4. Major neurologic or metabolic disorder or other illness leading to hypoventilation and hypercarbia

3.3 Randomization procedure

Following informed consent from the parent/guardian, the infants will be randomly assigned to the NO group or control (oxygen) group by the study coordinator using blocks of sealed envelopes. The allocation proportions for the NO and control group will be 1: 1.

3.4 Apparatus, protocols, and monitoring

Apparatus:

We have developed a "double-hood" model for the administration of NO via oxygen hood. The oxygen/NO/N_2_ blend is administered to inner hood and
suctioned out of outer hood (and inner hood via NO/NO_2_ sensors). The inner hood is made of Plexiglas (Olympic oxyhood; 10 inch diameter) and the outer hood is made of plastic sheeting (NOVA tenthouse oxygen tent; 12 x 12 x 10 inch).

100% oxygen (15 lpm) is blended with a NO/nitrogen mixture (800 ppm NO; balance N_2_) to yield a final concentration of 24 ppm NO, NO_2_ 0.25 ppm, 97 % oxygen, 3 % nitrogen. This becomes the flow into the inner hood, from which the neonate breathes.

A suction apparatus is attached to the outer hood which suctions the gases in the outer hood at approximately 4 lpm. The NO/NO_2_ sensor lead in the inner hood also suctions approximately 4 lpm. Both suction systems are attached to a vented connector attached to the wall suction which entrains room air and is regulated to a negative pressure of –9 to –10 cm H_2_0.

The concentration of NO in the inner hood with this apparatus is approximately 20 ppm, with NO_2_ 0.95 ppm, and 97 % oxygen. The concentrations in the outer hood are: NO 15-17 ppm, with NO_2_ 0.60-1.05 ppm, and 65-77 % oxygen.

On the outer surface of the hood, near the opening, the concentrations of NO are 0.45-0.55 ppm, with NO_2_ 0.00 ppm, and 22-35 % oxygen. Further away from the hood, the oxygen concentration is 21% (same as room air) and NO_2_ is undetectable (0.00 ppm). NO levels are < 0.25 ppm at more than 12 inches (30 cm) from the hood.

Protocols:

After entry criteria are met, informed consent is obtained from the parents by either the study coordinator, research nurse, or attending physician/fellow and the neonate will be randomized to either the NO group or control group. The treatment gas will be administered by hood, with the clinicians and nurses blinded to assignment. The apparatus setup at the bedside would be identical for both groups and the NO flowmeter and NO/NO_2_ concentrations will be masked. FiO_2_ and NO/NO_2_ concentrations of the inflow gas will be monitored at the beginning of the study period and the sensors will then be moved to the inner hood. Respiratory therapists will adjust flow and blending to administer 20 ppm of NO or an equivalent flow of O_2_. FiO_2_ and NO/NO_2_ concentrations in the inner hood will be monitored continuously for the study period. Physicians and nurses caring for the patient and analyzing results will be blinded to patient assignment. After completion of one hour on the study, the gas will be weaned every hour over the next four hours (20 ppm at 1 hour to 10 ppm at 2 hours to 5 ppm at 3 hours to 2.5 ppm to 4 hours to 0 ppm at 5 hours), then discontinued. If marked deterioration (decrease in oxygen saturation of > 5%) is seen during the weaning phase, the treatment gas can be increased to the previous level and can then be weaned subsequently by the same decrement but over two hours. The attending physician can initiate mechanical ventilation at any point during this study, based on clinical or other grounds. If methemoglobinemia (MetHb>5%) occurs, or if NO_2_ concentrations are >3% in the inner hood, or if oxygenation decreases significantly (decrease in oxygen saturation of > 5%), or if other adverse reactions that could possibly be related temporally to the treatment gas are evident, the gas is to be weaned in a shorter timespan (50% every 15 minutes). The results of these neonates will still be analyzed based on "intention to treat". If at the end of the study period the infant still meets entry criteria, he/she will be crossed over into the other treatment group.

Monitoring:

1. Apparatus: FiO_2_ and NO/NO_2_ concentrations will be checked in the **inflow gas** at the beginning, and continuous monitoring of FiO_2_ and NO/NO_2_ concentrations in the **inner hood** will be done till exit from study. FiO_2_ and NO/NO_2_ concentrations will also be measured **outside the hood** 30 min and one hour after commencement of the treatment, at a distance of one foot (30 cm) from the front of the hood.
2. Baby: **ABGs** at 0 hours (the last ABG before beginning treatment gas) and 1 hour will be used for the study. **MetHb** will also be measured in these samples. Frequency of ABG and other lab investigations at other times will be at the discretion of the attending physician, but should be at least q 1 hour for the five hours on the study. **Oxygen saturation, heart rate, respiratory rate** and **blood pressures** (by arterial catheter) will be monitored continuously through the period of study. A **platelet count** obtained less than 12 hours before entry into the study will also be compared with one obtained immediately after completion of one hour of exposure to the treatment gas. **Echocardiograms** are encouraged before entry into the study, as echocardiograms are usually part of the clinical investigation in neonates with suspected PPHN, but are not a pre-requisite for entry in view of time and technical constraints.

3.5 General care

Close attention to thermoregulation and fluid balance is recommended. Minimal handling and suctioning is recommended. Fluid and electrolyte management is left to the discretion of the attending physician, but data will be collected on total fluid intake, serum sodium levels, and sodium bicarbonate infusions.

3.6 Co-interventions and contamination

Co-interventions will be minimized by randomization, since all groups should be affected approximately equally. However, note will be made of interventions that could potentially alter outcome variables, such as fluid management. The major contaminant is that neonates with elevated A-a DO_2_ are a heterogeneous population with variable degrees of V/Q mismatch and right to left shunting and may not respond either in the same direction or magnitude to any intervention, including the administration of NO. However, this study is a pilot study to evaluate the effect of NO administered by oxygen hood in a "real-world" situation where heterogeneity is the rule, and transition from fetal to neonatal physiology is occurring. Another possible contaminant is that the NO group will have a slightly lower FiO_2_ (about 95-96% rather than 97-98% in the oxygen hood, due to admixture of the nitrogen carrier gas) than the oxygen group, and this will place the NO group at a disadvantage regarding the delivered oxygen concentration. However, similar small decreases in FiO_2_ occur even with NO use during mechanical ventilation and this is considered acceptable practice, since the improvements in oxygenation with NO are considered to outweigh the decreases due to the small reduction in FiO_2_.

3.7 Study exit criteria

1. Completion of one hour on treatment gas followed by weaning to 100% oxygen by hood. If at the end of the study period the infant still meets entry criteria (A-a DO_2_ 500- 600 mm Hg), he/she will be crossed over into the other treatment group.
2. If methemoglobinemia (MetHb>5%) occurs, or if NO_2_ concentrations are >3% in the inner hood, or if oxygenation decreases significantly (decrease in oxygen saturation of > 5%), or if other significant adverse reactions that could possibly be related temporally to the treatment gas are evident, the gas is to be weaned in a shorter timespan (50% every 15 minutes).
3. Sudden marked deterioration in any physiological variable, that in the opinion of the attending physician, requires initiation of mechanical ventilation or ECMO (initiation of inotropic support, change of intravenous fluid intake, or antibiotic therapy are not included in exit criteria).
4. Transfer to another institution / death / withdrawal from study by parent or attending physician

The study coordinator is to be contacted in cases where criteria 2, 3, or 4 are being considered.

3.8 Outcomes and definitions

1. PaO_2_: Partial pressure of oxygen in post-ductal arterial blood
2. A-a DO_2_ : calculated by PAO_2_ - PaO_2_

where PAO_2_ = [FiO_2_ x (PB - 47)] - PaCO_2_

PB= ambient barometric pressure in mm Hg

1. A/a O_2_ : calculated by PAO_2_ / PaO_2_

where PAO_2_ = [FiO_2_ x (PB - 47)] - PaCO_2_

1. SpO_2_: Oxygen saturation by pulse oximetry (pre-ductal)

3.9 Statistical issues

The power of the study is 85% with n=20 in each group, α=0.05, assuming we need to detect a difference of 25 % with a standard deviation at 25% of the mean. Repeated measures ANOVA or unpaired t-test will be used if data are normally distributed, otherwise non-parametric tests will be used. Data will be analyzed by intention to treat. Small numbers are required since this is a pilot study to show physiologic efficacy of the technique and is not meant to study clinical outcomes.

**4. Ethical considerations**

1. Is NO safe to administer by oxyhood? : 20 ppm of NO has been safely administered during mechanical ventilation to many neonates and infants over the last few years. Administration by oxygen hood, when the concentration of inhaled NO is being continuously monitored, is probably as safe or even safer than administration during mechanical ventilation, since the additional hazards of barotrauma/volutrauma of mechanical ventilation are not superimposed.
2. Is it ethical to withdraw NO after a one hour administration at 20 ppm? : Since this is a pilot study to evaluate the physiologic efficacy of the method, and is not an evaluation of long-term NO by oxyhood on clincal outcomes, it is ethical to wean off NO to 100% oxygen by hood, which is the established practice.
3. Is it ethical to use oxygen rather than nitrogen as the control gas, since the FiO_2_ of the oxygen group will be higher? : Oxygen is the established and conventional method, and is known to reduce pulmonary arterial pressures. Hence it is ethical to use oxygen rather than nitrogen as the control gas.
4. If weaning from treatment gas is difficult, should treatment be continued on a long term basis? : Again, since this study is to evaluate the physiologic efficacy and not clinical outcomes, and this method of administration is still experimental, we do not favor administration of the treatment gas on a long term basis, although slower weaning can be performed.
5. If a response is seen to the treatment gas, but is not marked, should the treatment gas concentration be increased?: No, most studies have shown that neonates who respond usually do so with 20 ppm. Also, with increasing treatment gas, FiO_2_ decreases. When the concentration is maintained at 20 ppm or less, FiO_2_ is more than 95%.
6. Is it ethical to use a control group, since NO is already known to reduce pulmonary pressures and improve oxygenation in mechanically ventilated animals and neonates? : Oxygen is the established and conventional method, and the administration of NO by hood is still experimental. Also, there is provision for a crossover to the other group if the neonate still meets entry criteria after weaning from the treatment gas, so in case of no improvement, the neonate will still access both forms of therapy.
7. **Feasibility**
   1. Available population / estimated recruitment time

Every week at UAB RNICU, approximately one term neonate requires administration of 100% oxygen by hood for an elevated A-a DO_2_. This works out to about 50 neonates per year. Based on the assumption that consent would be available in 80%, and one fourth of the remainder (10 of 40) are excluded for various reasons, such as inability to place an arterial catheter, rapid deterioration requiring mechanical ventilation, etc, 30 neonates can be enrolled in one year. The estimated recruitment time is hence 15 months for the projected sample size of 40. In case of slow enrollment over the first 3 months, the NICU at Children’s hospital and at Cooper Green can also be used for enrollment, after IRB approval.

5.2 Potential pitfalls of study design

One problem is the heterogeneity in patient population, with a varying combination of V/Q mismatch and right to left shunting of different etiologies. The response to the treatment gas may hence differ in direction and magnitude. Another issue is that a significant proportion of neonates may also improve very rapidly, before and during entry into the study. This should hopefully be equally divided among the groups by randomization.

**References:**

1. Wessel DL, Adatia I, Van Marter LJ, Thompson JE, Kane JW, Stark AR, Kourembanas S: Improved oxygenation in a randomized trial of inhaled nitric oxide for persistent pulmonary hypertension of the newborn. Pediatrics 1997; 100:e7 (http://www.pediatrics.org/cgi/content/full/100/5/e7)
2. Roberts JD, Fineman JR, Morin FC 3^rd^, Shaul PW, Rimar S, Schreiber MD, Polin RA, Zwass MS, Zayek MM, Gross I, Heymann MA, Zapol WM: Inhaled nitric oxide and persistent pulmonary hypertension of the newborn. The Inhaled Nitric Oxide Study Group. N Engl J Med 1997; 336: 605-610
3. The Neonatal Inhaled Nitric Oxide Study Group: inhaled nitric oxide in full-term and nearly full-term infants with hypoxic respiratory failure. N Engl J Med 1997; 336: 597-604
4. Truog WE: Inhaled nitric oxide: a tenth anniversary observation. Pediatrics 1998; 101: 696-697
5. Nicholas Nelson: The Onset of Respiration. Chap 11, Neonatology: Pathophysiology and Management of the Newborn. Ed. Gordon Avery, 3^rd^ Ed, JB Lippincott Co., 1987, pp 176-200
6. **Furchgott** RF: The 1996 Albert Lasker Medical Research Awards. The discovery of **endothelium-**derived relaxing factor and its importance in the identification of **nitric oxide**. JAMA 1996; 276(14):1186-1188
7. Roberts JD, Polaner DM, Lang P, Zapol WM: Inhaled nitric oxide in persistent pulmonary hypertension of the newborn. Lancet 1992; 340: 818-819
8. Kinsella JP, Neish SR, Shaffer E, Abman SH: Low-dose inhalation nitric oxide in persistent pulmonary hypertension of the newborn. Lancet 1992; 340: 819-820
9. Day RW, Lynch JM, White KS, Ward RM: Acute response to inhaled nitric oxide in newborns with respiratory failure and pulmonary hypertension. Pediatrics 1996; 98: 698-705
10. Finer NN, Etches PC, Kamstra B, Tierney AJ, Peiowski A, Ryan CA: Inhaled nitric oxide in infants referred for extracorporeal membrane oxygenation: dose response. J Pediatr 1994; 124: 302-308
11. Barefield ES, Karle VA, Philips JB 3^rd^, Carlo WA: Inhaled nitric oxide in term infants with hypoxemic respiratory failure. J Pediatr 1996; 129: 279-286
12. Kakuya F, Takase M, Ishii N, Kajino M, Hayashi T, Miyamoto K, Muraki S, Iwamoto J, Okuno A: Inhaled nitric oxide therapy via nasopharyngeal tube in an infant with end-stage pulmonary hypertension. Acta Paediatr Jpn 1998; 40: 155-158
13. Carlo WA, Martin RJ, Bruce EN, Strohl KP, Fanaroff AA: Alae nasi activation (nasal flaring) decreases nasal resistance in preterm infants. Pediatrics 1983; 72:338-343
14. Solow B, Pietersen B: Nasal airway resistance in the newborn. Rhinology 1991; 29: 27-33
15. Ivy DD, Griebel JL, Kinsella JP, Abman SH: Acute hemodynamic effects of pulsed delivery of low flow nasal nitric oxide in children with pulmonary hypertension [abstract]. Pediatr Res 1998; 43:23A
